# Prognostic Value of Right Ventricular Ejection Fraction Assessed by 3D Echocardiography in COVID-19 Patients

**DOI:** 10.3389/fcvm.2021.641088

**Published:** 2021-02-09

**Authors:** Yanting Zhang, Wei Sun, Chun Wu, Yiwei Zhang, Li Cui, Yuji Xie, Bin Wang, Lin He, Hongliang Yuan, Yongxing Zhang, Yu Cai, Meng Li, Yu Zhang, Yun Yang, Yuman Li, Jing Wang, Yali Yang, Qing Lv, Li Zhang, Mingxing Xie

**Affiliations:** ^1^Department of Ultrasound, Tongji Medical College, Union Hospital, Huazhong University of Science and Technology, Wuhan, China; ^2^Clinical Research Center for Medical Imaging in Hubei, Wuhan, China; ^3^Hubei Province Key Laboratory of Molecular Imaging, Wuhan, China

**Keywords:** three-dimensional echocardiography, right ventricular function, Coronavirus disease 2019, myocardial strain, prognosis

## Abstract

**Background:** RVEF (right ventricular ejection fraction) measured by three-dimensional echocardiography (3DE) has been used in evaluating right ventricular (RV) function and can provide useful prognostic information in other various cardiovascular diseases. However, the prognostic value of 3D-RVEF in coronavirus disease 2019 (COVID-19) remains unknown. We aimed to investigate whether 3D-RVEF can predict the mortality of COVID-19 patients.

**Methods:** A cohort of 128 COVID-19-confirmed patients who had undergone echocardiography were studied. Thirty-one healthy volunteers were also enrolled as controls. COVID-19 patients were divided into three subgroups (general, severe, and critical) according to COVID-19 severity-of-illness. Conventional RV structure and function parameters, RV free wall longitudinal strain (FWLS) and 3D-RVEF were acquired. RVFWLS was measured by two-dimensional speckle tracking echocardiography. RVEF was acquired by 3DE.

**Results:** Compared with controls, 2D-RVFWLS and 3D-RVEF were both significantly decreased in COVID-19 patients (−27.2 ± 4.4% vs. −22.9 ± 4.8%, *P* < 0.001; 53.7 ± 4.5% vs. 48.5 ± 5.8%, *P* < 0.001). Critical patients were more likely to have a higher incidence of acute cardiac injury and acute respiratory distress syndrome (ARDS), and worse prognosis than general and severe patients. The critical patients exhibited larger right-heart chambers, worse RV fractional area change (RVFAC), 2D-RVFWLS, and 3D-RVEF and higher proportion of pulmonary hypertension than general and severe patients. Eighteen patients died during a median follow-up of 91 days. The multivariate Cox regression analysis revealed the acute cardiac injury, ARDS, RVFAC, RVFWLS, and 3D-RVEF were independent predictors of death. 3D-RVEF (chi-square to improve 18.3; *P* < 0.001), RVFAC (chi-square to improve 4.5; *P* = 0.034) and 2D-RVFWLS (chi-square to improve 5.1; *P* = 0.024) all provided additional prognostic value of higher mortality over clinical risk factors. Moreover, the incremental predictive value of 3D-RVEF was significantly (*P* < 0.05) higher than RVFAC and RVFWLS.

**Conclusion:** 3D-RVEF was the most robust independent predictor of mortality in COVID-19 patients and provided a higher predictive value over conventional RV function parameters and RVFWLS, which may be helpful to identify COVID-19 patients at a higher risk of death.

## Introduction

Cardiac injury was a prevalent complication and was associated with worse prognosis in COVID-19 patients, with an incidence ranging from 7.2 to 27.8% (1–5). The increased cardiac workload resulting from respiratory failure and hypoxemia is a common mechanism of cardiac injury and the right ventricle may bear the brunt of its impact (3). Echocardiography is a convenient and widely available imaging tool for assessing cardiac function. Although both left ventricular (LV) dysfunction and right ventricular (RV) dysfunction are noted in hospitalized COVID-19 patients, the incidence of the latter is higher and the worse RV function is associated with clinical deterioration (i.e., hemodynamic instability, cardiac deterioration, and respiratory deterioration) (6–8). Furthermore, right ventricular free wall longitudinal strain (RVFWLS) derived from two-dimensional speckle tracking echocardiography (2D-STE) has been proven to be a more effective factor to predict mortality than conventional RV function parameters in COVID-19 patients (9). However, 2D-STE has the intrinsic limitation of losing speckles from out-of-plane cardiac motion. Additionally, given the complex structure of the RV and the three–dimensional (3D) motion of heart, 3D analysis could potentially provide better and more accurate assessment compared to 2D analysis. Previous studies have proved that three–dimensional right ventricular ejection fraction (3D-RVEF) can provide valuable prognostic information in various cardiovascular diseases (10–12). However, the prognostic value of 3D-RVEF in COVID-19 patients has not been studied. Accordingly, this study aimed to assess RV structure and function in COVID-19 patients with different severity of illness and to explore whether 3D-RVEF provides incremental prognostic value with regards to fatal outcomes in COVID-19 patients.

## Methods

### Study Population

This study was performed at Union Hospital in Wuhan, China. We enrolled a total of 172 consecutive patients confirmed with COVID-19 according to the WHO interim guidance (13) from January 29 to March 4, 2020. Bedside echocardiogram was performed in all patients for assessment of cardiac structure and function. The median time from admission to echocardiography examination was 5 days [interquartile range (IQR) 3–10 days]. A total of 44 patients were excluded because of dilated cardiomyopathy (*n* = 2), old myocardial infarction (*n* = 4), insufficient image quality for echocardiographic analysis (*n* = 32), arrhythmia during examination (*n* = 6), the remaining 128 patients were divided into three subgroups according to the guideline on the diagnosis and treatment of COVID-19 by the National Health Commission (version 7.0) (14): general (*n* = 41), severe (*n* = 58) and critical (*n* = 29) groups. Additionally, thirty-one healthy volunteers having no cardiopulmonary disease based on physical examinations, biochemical tests, electrocardiography, chest X-ray and echocardiogram were enrolled as the control group.

This study was approved by the Ethics Committee of Tongji Medical College, Huazhong University of Science and Technology. Written informed consent was waived for all participants with emerging infectious diseases.

### Clinical Data

The demographic characteristics and clinical data (vital signs, comorbidities, major laboratory findings, treatment, complications, and prognosis during hospitalization) were extracted from electronic medical records by two researchers. The timing of laboratory measurements was within 3 days of echocardiogram with a median interval of 1 day (Interquartile Range, IQR: 1–2 days). Patients clinical outcomes were followed up to May 18, 2020. Acute cardiac injury was defined as serum plasma levels of high-sensitivity troponin I (hs-TNI) above the 99th percentile of the upper limit of reference (4). Acute respiratory distress syndrome (ARDS) was defined according to the Berlin Definition (15). The criteria for COVID-19 severity-of-illness was defined by the Chinese management guideline for COVID-19 (version 7.0) as follows: (1) general: fever and respiratory symptoms, with evidence of pneumonia on radiological imaging; (2) severe: patients with any of the following symptoms and signs: respiratory distress with respiratory rate ≥30 breaths/min; SpO2 ≤ 93% at rest; and PaO2/FiO2 ≤ 300 mmHg (1 mm Hg = 0.133 kPa); and (3) critical: patients with any of the following conditions: respiratory failure requiring mechanical ventilation, shock, and/or other organ failure requiring admission to the intensive care unit (ICU) (14). The criteria for RV dysfunction is based on published reference, and the COVID-19 patients were divided into three subgroups: 3DRVEF > 45%, 40% < 3DRVEF ≤ 45%, and 30% < 3DRVEF ≤ 40% (16).

### Conventional Echocardiography

Bedside echocardiography was performed using a commercially available system (EPIQ 7C, Philips Medical Systems, Andover, USA). 2D and Doppler echocardiography examinations were performed based on the recommendations of the American Society of Echocardiography (17). And all 2D echocardiographic parameters were acquired according to the published guidelines (18, 19).

The left atrial volume, left ventricular end-diastolic and end-systolic volumes, left ventricular ejection fraction (LVEF) were measured by the biplane Simpson's method in apical two- and four-chamber views and volumes were indexed to body surface area (BSA) (18). Doppler mitral and tricuspid peak early (E) and late (A) diastolic velocities, and E/A velocity ratios were measured from the LV and RV inflow velocities on apical four-chamber view. RV transverse diameter at the base was measured from the RV-focused apical four-chamber view, and the minor right atrial (RA) transverse diameter was measured from the middle level of RA on apical four-chamber view. Tricuspid lateral annular systolic velocity (S'), tricuspid annular plane systolic excursion (TAPSE) and RV fractional area change (FAC) were measured according to the established guidelines (19). Systolic pulmonary arterial pressure (PASP) was calculated by the Bernoulli simplified equation on tricuspid regurgitation (TR) maximum jet velocity sum of estimated RA pressure. Pulmonary hypertension (PH) was defined as PASP > 40 mm Hg (19).

The off-line 2D-STE analysis was performed with the vendor-independent software TomTec (2D Cardiac Performance Analysis 1.2 for 2D-STE; TomTec Imaging Systems, Unterschleissheim, Germany) to acquire the RV strain in the RV focused apical four-chamber view with frame rate of 50–70 MHz, according to the published recommendations (20, 21). The workstation automatically performed a contour tracking of RV endocardium, and a manual adjustment was performed in case of unsatisfactory tacking. Finally, the time-strain curve of RVFWLS was generated automatically. RVFWLS was defined as the mean longitudinal peak systolic strain of three segments of the RV free wall. RVFWLS was performed 3 times during the regular heartbeats and the average was used for analysis.

### 3DE Imaging and Analysis

**Figure 1:**
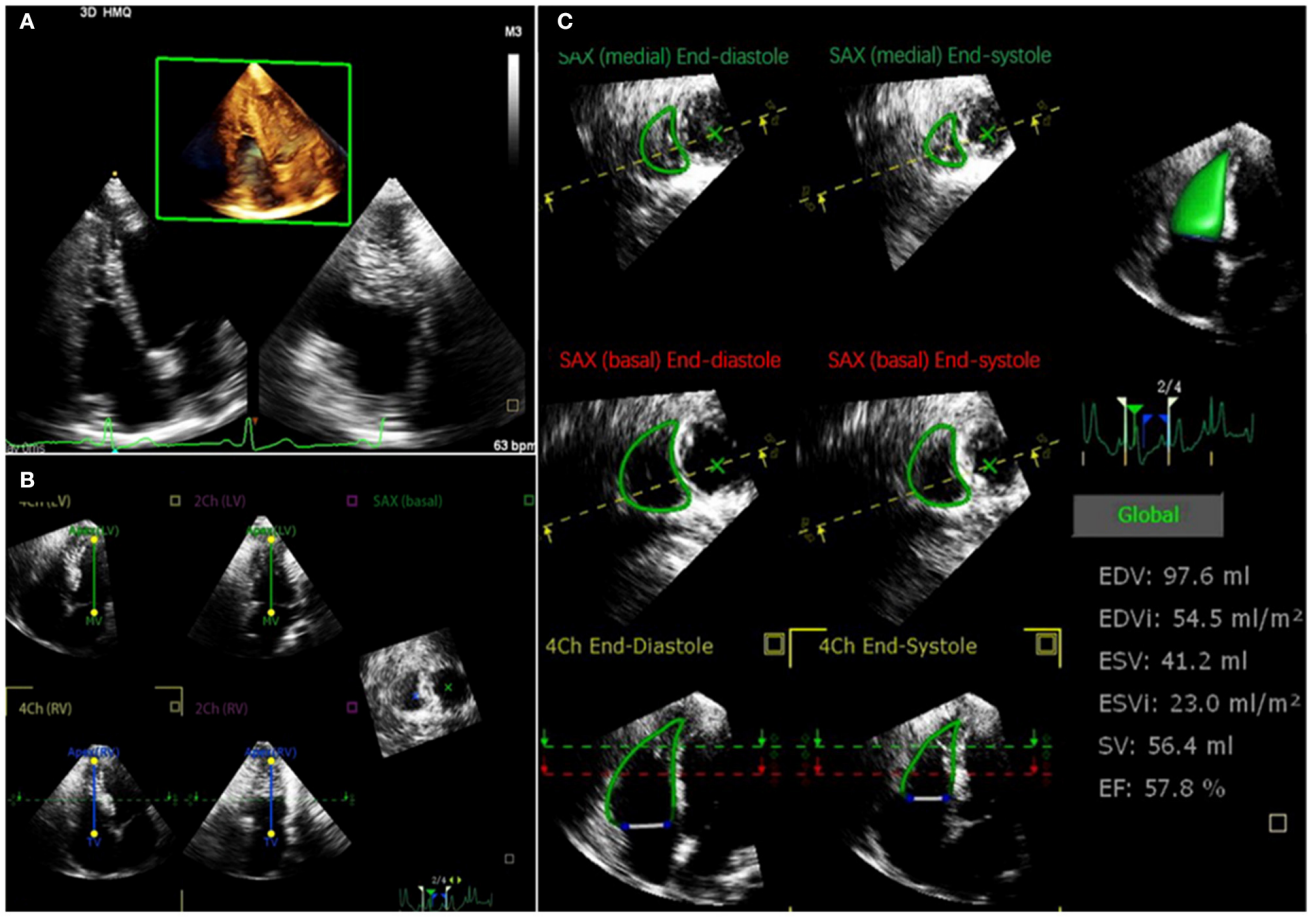
Representative 3DE analysis in a COVID-19 patient. **(A)** Retrieving 3DE datasets aimed for the RV full-volume images from apical 4-chamber RV-focused view; **(B)** LV and RV long-axis landmarks in end-diastole in the apical 2- and 4-chamber views were identified by software initially; then, RV focused 4-chamber view and a short-axis view are derived; **(C)** RV endocardial surfaces were full automatically defined and tracked throughout the cardiac cycle; finally, RV end-diastolic volume, RV end-systolic volume, and RVEF were determined. 3DE, three-dimensional echocardiography; COVID-19, coronavirus disease 2019; LV, left ventricular; RV, right ventricular; RVEF, right ventricular ejection fraction.

A wide-angled, single-beat, high frame rate (HeartModel mode) 3D full-volume images data sets were acquired from the apical 4-chamber RV-focused view. The 3DE datasets were stored digitally for offline analysis. The 3D full-volume RV images were analyzed by an experienced echocardiographer. RV-focused one-beat 3D full-volume images were analyzed with a novel, full automated RV quantification software (3D Auto RV, Phillips Medical Systems) that detect RV endocardial contours using artificial intelligence, which consists of knowledge-based identification of initial global shape and RV chamber orientation, followed by 3D speckle tracking analysis throughout a cardiac cycle (22, 23). The software initially identified LV and RV long-axis landmarks in end-diastole in the apical two- and four-chamber views. Based on that, the RV-focused four-chamber view and a short-axis view. Then RV endocardial surfaces were full automatically defined and tracked throughout the cardiac cycle, and a quick minimal manual adjustment was performed in case of unsatisfactory outcomes. Finally, a 3D RV cast, RV volume curves were provided, from which the RV end-diastolic volume (RVEDV), RV end-systolic volume (RVESV), and RVEF were determined (Figures 1A–C).

### Interobserver and Intraobserver Reproducibility

Intraobserver and interobserver variability in measurement of 2D-RVFWLS and 3D-RVEF were analyzed in 20 randomly selected subjects. Intraobserver variability was assessed by the same observer 2 weeks later. Interobserver variability was assessed by a second observer in the same 20 patients.

### Statistical Analysis

Continuous variables were expressed as mean ± SD, or median (IQR). The normality of distribution was tested by the Shapiro–Wilk test. Comparisons between groups were made by two-sample student *t*-test or one-way analysis of variance for normally distributed variables; and Mann–Whitney *U*-test or Kruskal–Wallis test for non-normal distribution of data. The *post-hoc* pairwise comparisons with Bonferroni correction was used for continuous variables. Categorical data were expressed as percentages and were compared by the χ^2^ test or Fisher exact test, when appropriate. The correlation between 3D-RVEF and 2D-RVFWLS was examined using Pearson's Correlation coefficients.

Univariate and multivariable Cox proportional hazards models were performed to identify the independent risk factors of mortality in COVID-19 patients. Variables with *P* < 0.05 at univariate analysis were included in stepwise multivariable analysis. To avoid overfitting and collinearity issues, four separate multivariable Cox proportional hazard models were constructed to determine the independent predictors of higher mortality. To assess the potential additive prognostic value of 3D-RVEF and the other RV parameters, we evaluated the additional increment of the chi-square statistics of the combined models over the baseline model. Receiver operator characteristic curves (ROC) were used to calculate the sensitivity and specificity for predicting death by RV function index and to determine the optimal prognostic cutoff value (Youden method). The Hanley and McNeil methods were applied for comparison of area under the curves (AUCs) of RV function parameters (24). Survival curves were obtained using the Kaplan–Meier method and compared by the log-rank test. The reproducibility of 2D- RVFWLS and 3D-RVEF was assessed using intra-class correlation coefficients (ICC) and Bland-Altman analyses.

All statistical analyses were performed using SPSS version 23.0 (Statistical Package for the Social Sciences, Chicago, IL, USA), STATA software version 10 (StataCorp, Texas, USA) and R version 3.6.3 (R Foundation for Statistical Computing, Vienna, Austria). All tests were 2-tailed; *P* < 0.05 was considered statistically significant.

## Results

### Clinical Characteristics

The clinical characteristics of the 128 COVID-19 patients were shown in Table 1. The mean age was 61.3 ± 13.1 years and 61 (47.7%) patients were men. Of 128 patients, 7 (5.5%) had chronic obstructive pulmonary disease, 18 (14.1%) had cardiac disease including 14 with known coronary heart disease in the absence of abnormal wall motion by routine echocardiography and 4 with occasional arrhythmia (atrial and ventricular extrasystole) by the recording of a long-term electrocardiograph. Compared with general and severe patients, critical patients were older, predominantly male and had higher heart rates (HR) and lower oxygenation index.

**Table 1:** Clinical characteristics of COVID-19 patients according to severity of illness.

**Variables**	**Total (*n* = 128)**	**General (*n* = 41)**	**Severe (*n* = 58)**	**Critical (*n* = 29)**	***P*-value**
**Clinical characteristics**					
Age (years)	61.3 ± 13.1	58.6 ± 16.0	60.9 ± 11.7	66.0 ± 9.8	0.06
Male, *n* (%)	61 (47.7)	15 (36.6)	26 (44.8)	20 (69.0)	0.024
Heart rate, beats/min	86 (80, 99)	84.0 (80, 95)	89 (80, 101)	90 (80, 99)	0.494
Respiratory rate, times/min	23 (20, 30)	20 (20, 23)	25 (20, 30)	26 (20, 33)	<0.001
SBP, mmHg	130 (120, 140)	132 (122, 146)	125 (116, 138)	134 (120, 146)	0.195
DBP, mmHg	80 (73, 88)	81 (75, 90)	78 (72, 87)	80 (74, 87)	0.325
OI, mmHg	286.0 (200.0, 337.9)	340.7 (317.2, 392.0)	250.7 (205.5, 301.7)	173.0 (141.7, 248.4)	<0.001
**Comorbidities**					
Hypertension, *n* (%)	52 (40.6)	17 (41.5)	20 (34.5)	15 (51.7)	0.301
Diabetes, *n* (%)	18 (14.1)	7 (17.1)	8 (13.8)	3 (10.3)	0.720
Cardiac disease, *n* (%)	18 (14.1)	4 (9.8)	5 (8.6)	9 (31.0)	0.028
COPD, *n* (%)	7 (5.5)	3 (7.3)	2 (3.4)	2 (6.9)	0.684
Chronic liver diseases, *n* (%)	4 (3.1)	1 (2.4)	3 (5.2)	0 (0.0)	0.559
Chronic kidney disease, *n* (%)	1 (0.8)	0 (0.0)	1 (1.7)	0 (0.0)	1.000
Malignancy, *n* (%)	9 (7.0)	2 (4.9)	5 (8.6)	2 (6.9)	0.904
Smoker, *n* (%)	7 (5.5)	4 (9.8)	3 (5.2)	0 (0.0)	0.235
**Laboratory findings**					
White blood cell × 10^9^/L	6.6 (4.9, 9.4)	6.2 (4.5, 9.3)	6.0 (4.8, 8.7)	8.4 (6.2, 10.8)	0.010
Lymphocyte count × 10^9^/L	1.01 (0.61, 1.44)	1.28 (1.00, 1.63)	0.97 (0.67, 1.36)	0.60 (0.28, 1.02)	<0.001
CRP, mg/L	26.3 (3.6, 63.3)	3.7 (1.0, 32.8)	24.0 (8.4, 53.3)	77.6 (49.0, 124.5)	<0.001
PCT, ng/ml	0.08 (0.05, 0.20)	0.06 (0.04, 0.17)	0.08 (0.05, 0.18)	0.15 (0.07, 0.32)	0.015
D-dimer, mg/L	1.4 (0.5, 5.8)	1.0 (0.2, 4.2)	1.2 (0.5, 5.8)	2.5 (1.0, 8.0)	0.006
hs-TNI, ng/mL	3.9 (1.8, 19.9)	2.7 (1.3, 12.1)	3.4 (1.6, 8.7)	29.3 (4.7, 74.8)	<0.001
CK-MB, U/L	12.0 (9.0, 20.8)	10.0 (7.0, 15.5)	12.0 (9.0, 16.0)	26.0 (11.0, 31.0)	0.001
BNP, pg/ml	51.8 (13.6, 140.9)	36.6 (10.0, 119.6)	33.5 (12.7, 83.1)	199.4 (102.5, 348.7)	<0.001
Serum creatinine (μmol/L)	65.1 (53.4, 80.5)	62.7 (49.3, 80.6)	63.3 (53.9, 82.0)	72.0 (59.3, 78.1)	0.235
**Treatments**					
Antiviral therapy, *n* (%)	120 (93.8)	34 (82.9)	57 (98.3)	29 (100.0)	0.004
Antibiotic therapy, *n* (%)	92 (71.9)	25 (61.0)	40 (69.0)	27 (93.1)	0.010
Glucocorticoid therapy, *n* (%)	50 (39.1)	11 (26.8)	19 (32.8)	20 (69.0)	0.001
ACE-I/ARB, *n* (%)	12 (9.4)	4 (9.8)	6 (10.3)	2 (6.9)	0.929
High-flow oxygen, *n* (%)	66 (51.6)	4 (9.8)	34 (58.6)	28 (96.6)	<0.001
Mechanical ventilation, *n* (%)	26 (20.3)	1 (2.4)	5 (8.6)	20 (60.9)	<0.001
IMV, *n* (%)	17 (13.3)	0 (0.0)	4 (6.9)	13 (44.8)	<0.001
NIMV, *n* (%)	9 (7.0)	1 (2.4)	1 (1.7)	7 (24.1)	0.001
ICU admission, *n* (%)	19 (14.8)	0 (0.0)	4 (6.9)	15 (51.7)	<0.001
**Complications**					
ARDS, *n* (%)	48 (37.5)	0 (0.0)	19 (32.8)	29 (100.0)	<0.001
Acute cardiac injury, *n* (%)	27 (21.1)	7 (17.1)	7 (12.1)	13 (44.8)	0.001
Acute kidney injury, *n* (%)	15 (11.7)	4 (9.8)	5 (8.6)	6 (20.7)	0.260
Coagulation dysfunction, *n* (%)	33 (25.8)	5 (12.2)	13 (22.4)	15 (51.7)	0.001
**Prognosis**					<0.001
Discharge, *n* (%)	110 (85.9)	41 (100.0)	56 (96.6)	13 (44.8)	
Death, *n* (%)	18 (14.1)	0 (0.0)	2 (3.4)	16 (55.2)	

*Data were n (%), mean ± SD or median (IQR). ARDS, acute respiratory distress syndrome; ACE-I, angiotensin-converting enzyme inhibitors; ARB, angiotensin II receptor blockers; BNP, B-type natriuretic peptide; CK-MB, creatine kinase muscle-brain; COVID-19, coronavirus disease 2019; COPD, chronic obstructive pulmonary disease; CRP, C-reactive protein; DBP, diastolic blood pressure; hs-TNI, high-sensitivity troponin I; ICU, intensive care unit; IMV, invasive mechanical ventilation; IQR; interquartile range; NIMV, non-invasive mechanical ventilation; OI, oxygenation index; PCT, procalcitonin; SBP, systolic blood pressure; SD, standard deviation*.

In addition, compared with general and severe patients, critical patients were more likely to have underlying cardiac disease, lower levels of lymphocyte counts, higher levels of C-reactive protein and procalcitonin. They were also more prone to receive high-flow oxygen and invasive mechanical ventilation therapy, and were more likely to develop acute cardiac injury, ARDS. More often than not they got admitted to ICU, and had higher mortality.

### Echocardiographic Characteristics

Table 2 revealed the echocardiographic characteristics of the subjects. Compared with healthy controls, COVID-19 patients had thickened interventricular septum thickness (IVST), decreased mitral and tricuspid E/A, lower LVEF and FAC, and higher left ventricular end systolic volume index (LVESVI). 2D-RVFWLS and 3D-RVEF were both significantly lower in COVID-19 patients than in controls (−22.9 ± 4.8% vs. −27.2 ± 4.4%, *P* < 0.001; 48.5 ± 5.8% vs. 53.7 ± 4.5%, *P* < 0.001). Moreover, 3D-RVEF correlated significantly with 2D-RVFWLS in COVID-19 patients (*r* = −0.59, *P* < 0.001) and in controls (*r* = −0.64, *P* < 0.001). Furthermore, critical patients exhibited significantly higher mitral E/e′, larger RA, RV and pulmonary artery (PA) diameter, worse FAC, 2D-RVFWLS, and 3D-RVEF. Moreover, a higher proportion of critical patients had PH. Additionally, Table 2 showed that age, HR and systolic blood pressure (SBP) were significantly different between controls and COVID-19 patients. The echocardiographic parameters were further compared between controls and COVID-19 patients after making statistical adjustment of age, HR, and SBP in Table 3. After the adjustment, the differences between COVID-19 patients and controls persisted for the IVST, tricuspid E/A, LVESVI, LVEF, RVFAC, 2D-RVFWLS, and 3D-RVEF. Likewise, sex and BSA were significantly different among the general, severe and critical groups. So, the echocardiographic parameters among the three groups were further compared after statistical adjustment of sex and BSA in Table 3. Larger right heart chambers, worse RVFAC, 2D-RVFWLS, and 3D-RVEF remained statistically significant in critical patients than general and severe patients (Table 3).

**Table 2:** Comparisons of baseline characteristics and echocardiographic characteristics in healthy controls and COVID-19 patients.

				**COVID-19 patients**			
**Variables**	**Control (*n* = 31)**	**All patients (*n* = 128)**	***P*-value**	**General (*n* = 41)**	**Severe (*n* = 58)**	**Critical (*n* = 29)**	***P*-value**
Age (years)	51.5 ± 8.4	61.3 ± 13.1	<0.001	58.6 ± 16.0	60.9 ± 11.7	66.0 ± 9.8	0.060
Male, *n* (%)	18 (58.1)	61 (47.7)	0.298	15 (36.6)	26 (44.8)^*^	20 (69.0)^*^^#^	0.024
Body surface area, m^2^	1.69 ± 0.13	1.67 ± 0.15	0.589	1.66 ± 0.15	1.65 ± 0.15	1.76 ± 0.14^*^^#^	0.003
Heart rate, beats/min	66.0 (58.0, 73.0)	86.0 (80.0, 99.0)	<0.001	84.0 (80.0, 95.0)	89.0 (79.8, 101.3)	90.0 (80.0, 98.5)	0.494
SBP, mmHg	120.0 (114.0, 123.0)	130.0 (120.0, 140.0)	0.001	132 (122.5, 145.5)	124.5 (115.8, 138.0)	134.0 (120.0, 145.5)	0.195
DBP, mmHg	78.0 (70.0, 86.0)	80.0 (73.0, 87.8)	0.336	81.0 (74.5, 89.5)	77.5 (71.5, 87.3)	80.0 (73.5, 87.0)	0.325
**Left chamber**							
LA, mm	33.7 ± 3.3	34.3 ± 4.7	0.639	33.3 ± 4.9	34.2 ± 4.3	35.7 ± 5.1	0.087
LV, mm	46.9 ± 3.2	45.8 ± 4.3	0.101	44.8 ± 4.4	46.3 ± 3.8	46.2 ± 5.0	0.198
IVST, mm	8.9 ± 0.7	9.6 ± 1.2	0.001	9.6 ± 1.5	9.7 ± 1.0	9.5 ± 1.2	0.821
**Mitral valve**							
E/A	1.15 ± 0.31	0.93 ± 0.33	<0.001	0.9 ± 0.3	1.0 ± 0.4	0.9 ± 0.3	0.229
E/e'	7.9 ± 1.6	9.0 ± 3.0	0.144	8.1 ± 3.0	9.2 ± 3.0	9.8 ± 2.8^*^	0.004
LAVI, mL/m^2^	32.6 ± 9.1	34.2 ± 10.3	0.407	33.0 ± 10.8	35.5 ± 10.0	32.9 ± 10.7	0.313
LVEDVI, mL/m^2^	52.2 ± 12.4	54.5 ± 15.8	0.577	51.0 ± 17.5	57.8 ± 15.3^*^	52.4 ± 13.2	0.043
LVESVI, mL/m^2^	16.6 ± 4.0	20.0 ± 7.2	0.027	18.3 ± 7.3	21.8 ± 7.5^*^	18.8 ± 5.9	0.030
LVEF, %	68.1 ± 4.0	63.4 ± 6.2	<0.001	64.3 ± 4.8	62.5 ± 7.0	64.1 ± 6.3	0.295
**Right chamber**							
RA, mm	36.3 ± 3.9	35.3 ± 4.3	0.136	34.5 ± 3.5	34.3 ± 3.7	38.1 ± 5.1^*^^#^	<0.001
RV, mm	33.3 ± 3.5	33.9 ± 3.9	0.437	33.3 ± 3.4	33.3 ± 3.8	36.1 ± 4.2^*^^#^	0.004
PA, mm	23.3 ± 2.5	23.4 ± 2.7	0.752	22.1 ± 2.4	23.4 ± 2.5	25.1 ± 2.8^*^	<0.001
**Tricuspid valve**							
E/A	1.3 ± 0.2	1.0 ± 0.3	<0.001	1.0 ± 0.3	1.0 ± 0.3	0.9 ± 0.3	0.416
E/e'	5.1 ± 2.0	5.2 ± 1.8	0.343	5.2 ± 1.7	4.9 ± 1.5	5.8 ± 2.0	0.077
TAPSE, mm	24.0 ± 2.4	22.9 ± 3.8	0.169	22.9 ± 4.0	23.1 ± 3.5	22.3 ± 4.1	0.652
S′, cm/s	12.8 ± 2.0	14.1 ± 2.9	0.019	13.2 ± 2.1	14.2 ± 2.6	15.1 ± 3.9	0.117
FAC, %	51.2 ± 4.3	47.4 ± 5.7	<0.001	48.1 ± 5.2	46.8 ± 5.5	43.1 ± 5.0^*^^#^	0.001
PASP, mmHg	/	33.3 ± 12.8	/	27.0 ± 6.5	30.1 ± 8.9	45.3 ± 15.3^*^^#^	<0.001
PH, n (%)	0 (0)	18 (14.1)	0.025	1 (2.4)	4 (6.9)	13 (44.8)^*^^#^	<0.001
**2D-STE parameter**							
RVFWLS, %	−27.2 ± 4.4	−22.9 ± 4.8	<0.001	−23.9 ± 3.9	−24.2 ± 4.8	−19.1 ± 4.1^*^^#^	<0.001
**3DE parameters**							
RVEDVI, mL/m^2^	60.5 ± 12.9	61.8 ± 11.5	0.445	59.2 ± 10.9	61.2 ± 11.7	66.7 ± 10.8^*^^#^	0.036
RVESVI, mL/m^2^	28.0 ± 7.0	32.0 ± 7.6	0.005	28.9 ± 6.8	31.3 ± 6.5	37.8 ± 8.0^*^	<0.001
RVEF, %	53.7 ± 4.5	48.5 ± 5.8	<0.001	51.3 ± 5.6	48.9 ± 4.1	43.5 ± 5.8^*^^#^	<0.001

*Data were n (%), mean ± SD or median (IQR)*.

* *P < 0.05, vs. general group*;

# *P < 0.05, vs. severe groups*.

*DBP, diastolic blood pressure; RVFAC, right ventricular fractional area change; IVST, interventricular septum thickness; LA, left atrial diameter; LAVI, left atrial volume index; LV, left ventricular anteroposterior diameter; LVEDVI, left ventricular end-diastolic volume index; LVESVI, left ventricular end-systolic volume index; EF, ejection fraction; PA, pulmonary artery diameter; PASP, pulmonary artery systolic pressure; PH, pulmonary hypertension; RA, right atrial diameter; RV, right ventricular diameter; RVEDVI, right ventricular end-diastolic volume index; RVESVI, right ventricular end-systolic volume index; RVFWLS, right ventricular free wall longitudinal strain; S′, pulsed doppler peak velocity at the tricuspid lateral annulus; SBP, systolic blood pressure; STE, two-dimensional speckle tracking echocardiography; TAPSE, tricuspid annular plane systolic excursion; 2D, two-dimensional; 3DE, three-dimensional echocardiography*.

**Table 3:** Adjusted comparisons of echocardiographic characteristics in healthy controls and COVID-19 patients.

				**COVID-19 patients**			
**Variables**	**Control (*n* = 31)**	**All patients (*n* = 128)**	***P*-value**	**General (*n* = 41)**	**Severe (*n* = 58)**	**Critical (*n* = 29)**	***P*-value**
**Left chamber**							
LA, mm	34.1 (32.2, 35.9)	34.2(33.4, 35.0)	0.890	33.6 (32.1, 35.0)	34.4 (33.2, 35.6)	35.2 (33.4, 36.9)	0.378
LV, mm	46.4 (44.7, 48.1)	45.9 (45.2, 46.7)	0.651	45.0 (43.7, 46.3)	46.6 (45.5, 47.6)	45.3 (43.8, 46.8)	0.122
IVST, mm	9.0 (8.6, 9.5)	9.6 (9.4, 9.8)	0.036	9.7 (9.3, 10.1)	9.7 (9.4, 10.1)	9.3 (8.9, 9.8)	0.319
**Mitral valve**							
E/A	1.0 (0.9, 1.1)	1.0 (0.9, 1.0)	0.785	0.8 (0.7, 1.0)	1.0 (0.9, 1.1)	0.9 (0.8, 1.1)	0.199
E/e'	9.0 (7.8, 10.2)	8.7 (8.2, 9.2)	0.623	7.7 (6.8, 8.6)	9.2 (8.5, 10.0)^*^	10.0 (8.8, 11.1)^*^	0.007
LAVI, mL/m^2^	30.8 (26.5, 35.1)	34.6 (32.7, 36.6)	0.128	33.0 (29.5, 36.5)	35.6 (32.8, 38.4)	32.7 (28.6, 36.9)	0.396
LVEDVI, mL/m^2^	49.3 (42.9, 55.8)	55.2 (52.3, 58.1)	0.124	51.3 (46.3, 56.3)	57.1 (53.0, 61.2)	53.4 (47.3, 59.5)	0.193
LVESVI, mL/m^2^	15.1 (12.3, 18.0)	20.4 (19.2, 21.7)	0.002	18.3 (16.0, 20.6)	21.5 (19.6, 23.4)	19.4 (16.7, 22.2)	0.097
LVEF, %	68.6 (66.1, 71.1)	63.3 (62.2, 64.4)	<0.001	64.4 (62.3, 66.4)	62.6 (60.9, 64.3)	63.8 (61.3, 66.3)	0.386
**Right chamber**							
RA, mm	36.1 (34.4, 37.9)	35.3 (34.5, 36.1)	0.430	34.8 (33.6, 36.0)	34.4 (33.3, 35.4)	37.6 (36.1, 39.1)^*^^#^	0.003
RV, mm	32.5(30.9, 34.1)	34.1 (33.4, 34.8)	0.081	33.5 (32.4, 34.7)	33.4 (32.4, 34.4)	35.6 (34.2, 37.1)^#^	0.031
PA, mm	23.6 (22.5, 24.8)	23.3 (22.8, 23.8)	0.618	22.1 (21.3, 22.9)	23.6 (22.9, 24.2)^*^	24.8 (23.8, 25.8)^*^	<0.001
**Tricuspid valve**							
E/A	1.2 (1.1, 1.4)	1.0 (1.0, 1.1)	0.011	1.0 (0.9, 1.1)	1.0 (1.0, 1.1)	0.9 (0.8, 1.1)	0.371
E/e'	5.2 (4.4, 6.0)	5.2 (4.8, 5.6)	0.906	5.3 (4.6, 5.9)	4.9 (4.4, 5.4)	5.7 (5.0, 6.4)	0.199
TAPSE, mm	23.6 (22.1, 25.1)	23.0 (22.3, 23.6)	0.467	23.0 (21.8, 24.2)	23.1 (22.1,24.1)	22.1 (20.7, 23.6)	0.544
S′, cm/s	13.4 (12.2, 14.5)	13.9 (13.4, 14.4)	0.438	13.1 (12.2, 14.0)	14.2 (13.5,15.0)	15.1 (14.0, 16.2)^*^	0.019
RVFAC, %	50.1 (47.9, 52.3)	46.7 (45.7, 47.7)	0.010	48.2 (46.5, 49.9)	46.9 (45.5,48.3)	43.0 (40.9, 45.0)^*^^#^	0.001
PASP, mmHg	/	33.3 (30.1, 36.4)	/	27.0 (22.3, 31.8)	31.1 (27.2,35.0)	43.6 (38.5, 48.7)^*^^#^	<0.001
PH, n (%)	/	18 (14.1)	/	1 (2.4)	4 (6.9)	13 (44.8)^*^^#^	<0.001
**2D-STE parameter**							
RVFWLS, %	−26.0 (−24.0, −28.0)	−23.2 (−22.4, −24.1)	0.021	−23.9 (−22.5, −25.2)	−24.2 (−23.1, −25.4)	−19.1 (−17.4, −20.8)^*^^#^	<0.001
**3DE parameters**							
RVEDVI, mL/m^2^	57.6 (53.7, 62.5)	62.5 (60.4, 64.6)	0.092	59.6 (56.1, 63.0)	60.7 (57.9, 63.6)	67.1 (62.9, 71.3)^*^^#^	0.019
RVESVI, mL/m^2^	27.1 (23.9,30.2)	32.2 (30.9, 33.6)	0.006	29.2 (27.1, 31.3)	31.0 (29.3, 32.8)	37.9 (35.3, 40.5)^*^^#^	<0.001
RVEF, %	52.7 (50.3, 55.0)	48.7 (47.7, 49.7)	0.004	51.2 (49.7, 52.8)	48.9 (47.6, 50.2)	43.6 (41.7, 45.5)^*^^#^	<0.001

*Data were mean (95% CI)*.

* *P < 0.05, vs. general group*;

# *P < 0.05, vs. severe group*.

*FAC, right ventricular fractional area change; IVST, interventricular septum thickness; LA, left atrial diameter; LAVI, left atrial volume index; LV, left ventricular anteroposterior diameter; LVEDVI, left ventricular end-diastolic volume index; LVESVI, left ventricular end-systolic volume index; EF, ejection fraction; PA, pulmonary artery diameter; PASP, pulmonary artery systolic pressure; PH, pulmonary hypertension; RA, right atrial diameter; RV, right ventricular diameter; RVEDVI, right ventricular end-diastolic volume index; RVESVI, right ventricular end-systolic volume index; RVFWLS, right ventricular free wall longitudinal strain; S′, pulsed doppler peak velocity at the tricuspid lateral annulus; STE, speckle tracking echocardiography; TAPSE, tricuspid annular plane systolic excursion; 2D, two-dimensional; 3DE, three-dimensional echocardiography. Comparison of COVID-19 patients and controls adjusted for age, heart rate and systolic blood pressure. Comparison of COVID-19 patients with different severity of illness adjusted for sex and body surface area*.

During a median follow-up of 91 days (IQR: 74–93 days), 18 (14.1%) patients died. Non-survivors were more often male. They had lower oxygenation index than the survivors. The prevalence of comorbidities was similar between the two groups. Compared with non-survivors, survivors presented with more abnormal laboratory findings including lower lymphocyte, higher inflammation-related indices (white blood cell counts, C-reactive protein, procalcitonin, D-dimer) and elevated cardiac indices. There were no differences between the survivors and non-survivors in left heart chamber size and LV function parameters. However, the non-survivors showed larger RA, RV and PA diameters, lower tricuspid E/A, RVFAC, 2D-RVFWLS and 3D-RVEF than survivors. Moreover, a higher proportion of non-survivors presented PH than survivors (Table 4).

**Table 4:** Clinical and echocardiographic characteristics in COVID-19 survivors and non-survivors.

**Variables**	**Survivors (*n* = 110)**	**Non-survivors (*n* = 18)**	***P*-value**
**Clinical characteristics**			
Age (years)	61 ± 13	66 ± 12	0.106
Male, *n* (%)	48 (43.6)	13 (72.2)	0.024
Heart rate, beats/min	86 (80, 99)	90 (79, 114)	0.541
Respiratory rate, times/min	22 (20, 30)	30 (22, 36)	0.009
SBP, mmHg	130 (120, 140)	131 (119, 151)	0.790
DBP, mmHg	80 (73, 89)	79 (72, 81)	0.296
OI, mmHg	300.0 (217.4, 340.0)	195.1 (160.6, 240.2)	<0.001
**Comorbidities**			
Hypertension, *n* (%)	42 (38.2)	10 (55.6)	0.164
Diabetes, *n* (%)	16 (14.5)	2 (11.1)	1.000
Cardiac disease, *n* (%)	13 (11.8)	5 (27.8)	0.134
COPD, *n* (%)	5 (4.5)	2 (11.1)	0.255
Chronic liver diseases, *n* (%)	4 (3.6)	0 (0.0)	1.000
Chronic kidney disease, *n* (%)	1 (0.9)	0 (0.0)	1.000
Malignancy, *n* (%)	7 (6.4)	2 (11.1)	0.613
Smoker, *n* (%)	6 (5.5)	1 (5.6)	1.000
**Laboratory findings**			
White blood cell × 10^9^/L	6.2 (4.8, 8.9)	10.1 (7.2, 11.2)	0.001
Lymphocyte count × 10^9^/L	1.07 (0.70, 1.47)	0.45 (0.25, 0.69)	<0.001
CRP, mg/L	20.9 (2.9, 53.1)	79.1 (49.0, 129.9)	<0.001
PCT, ng/ml	0.07 (0.04, 0.16)	0.22 (0.09, 0.44)	0.001
D-dimer, mg/L	1.4 (0.5, 5.6)	2.2 (0.9, 8.0)	0.067
hs-TNI, ng/mL	3.3 (1.6, 8.7)	40.2 (17.4, 464.2)	<0.001
CK-MB, U/L	11.0 (8.0, 18.0)	21.0 (11.8, 35.3)	0.005
BNP, pg/ml	35.0 (10.0, 107.2)	207.4 (110.4, 525.2)	<0.001
Serum creatinine (μmol/L)	63.8 (53.5, 79.9)	72.7 (52.9, 87.0)	0.196
**Echocardiographic characteristics**			
**Left chamber**			
LA, mm	34.2 ± 4.5	35.1 ± 5.9	0.536
LV, mm	45.9 ± 4.3	45.0 ± 4.0	0.399
IVST, mm	9.7 ± 1.2	9.5 ± 1.1	0.592
**Mitral valve**			
E/A	0.9 ± 0.3	1.0 ± 0.4	0.663
E/e'	8.9 ± 3.0	9.6 ± 2.8	0.198
LAVI, mL/m^2^	34.3 ± 10.2	33.5 ± 11.7	0.696
LVEDVI, mL/m^2^	54.9 ± 16.4	51.6 ± 11.9	0.500
LVESVI, mL/m^2^	20.3 ± 7.4	18.3 ± 6.2	0.349
LVEF, %	63.1 ± 6.1	64.9 ± 6.8	0.280
**Right chamber**			
RA, mm	34.8 ± 3.6	38.2 ± 6.3	0.039
RV, mm	33.6 ± 3.7	36.3 ± 4.8	0.022
PA, mm	23.1 ± 2.6	25.0 ± 2.9	0.010
**Tricuspid valve**			
E/A	1.0 ± 0.3	0.9 ± 0.3	0.039
E/e'	5.2 ± 1.8	5.5 ± 1.5	0.173
TAPSE, mm	22.9 ± 3.8	22.3 ± 3.8	0.534
S′, cm/s	13.9 ± 2.6	15.1 ± 4.3	0.394
RVFAC, %	47.2 ± 5.2	41.6 ± 5.3	<0.001
PASP, mmHg	30.3 ± 9.6	45.7 ± 16.7	0.003
PH, *n* (%)	9 (8.2)	9 (50.0)	<0.001
RVFWLS, %	−23.7 ± 4.6	−18.3 ± 3.5	<0.001
RVEDVI, mL/m^2^	61.1 ± 11.3	66.4 ± 12.2	0.070
RVESVI, mL/m^2^	30.7 ± 6.6	39.8 ± 8.9	<0.001
RVEF, %	49.8 ± 4.8	40.4 ± 4.7	<0.001

*Data were mean ± SD, or n (%). BNP, B-type natriuretic peptide; CK-MB, creatine kinase muscle-brain; COPD, chronic obstructive pulmonary disease; COVID-19, coronavirus disease 2019; CRP, C-reactive protein; DBP, diastolic blood pressure; FAC, right ventricular fractional area change; hs-TNI, high-sensitivity troponin I; IVST, interventricular septum thickness; LA, left atrial diameter; LAVI, left atrial volume index; LV, left ventricular anteroposterior diameter; LVEDVI, left ventricular end-diastolic volume index; LVESVI, left ventricular end-systolic volume index; EF, ejection fraction; PA, pulmonary artery diameter; OI, oxygenation index; PASP, pulmonary artery systolic pressure; PCT, procalcitonin; PH, pulmonary hypertension; RA, right atrial diameter; RV, right ventricular diameter; RVEDVI, right ventricular end-diastolic volume index; RVESVI, right ventricular end-systolic volume index; RVFWLS, right ventricular free wall longitudinal strain; S′, pulsed doppler peak velocity at the tricuspid lateral annulus; SBP, systolic blood pressure; STE, speckle tracking echocardiography; TAPSE, tricuspid annular plane systolic excursion; 2D, two-dimensional; 3DE, three-dimensional echocardiography*.

### Prediction of the Death

**Figure 2:**
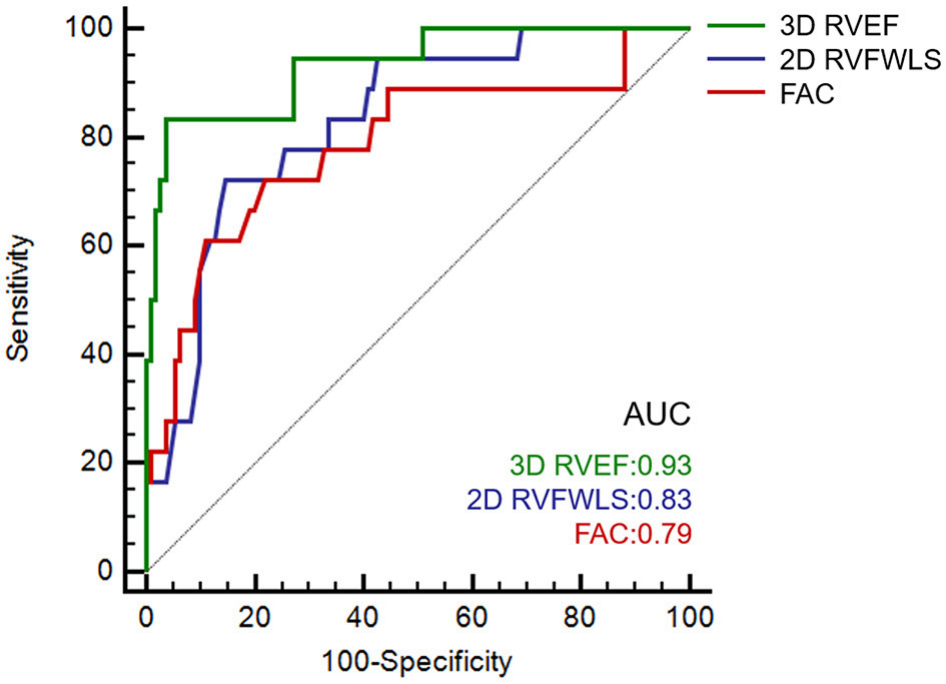
Receiver operating characteristic curves in predicting the death of COVIID-19 patients. COVID-19, coronavirus disease 2019.

Conventional RV function parameters including RVFAC, TAPSE and S′, 2D-RVFWLS and 3D-RVEF were analyzed by ROC for predicting mortality in COVID-19 patients. The ROC analyses showed only RVFAC, 2D-RVFWLS, and 3D-RVEF were associated with mortality (Figure 2). Moreover, the AUC of 3D-RVEF was greater than that of RVFAC (0.93 vs. 0.79, *P* = 0.039) and RVFWLS (0.93 vs. 0.83, *P* = 0.032). The best cutoff value to predict mortality was 42.7% for RVFAC (AUC, 0.79, *P* < 0.001; sensitivity, 72%; specificity, 78%), −18.9% for 2D-FWLS (AUC, 0.83, *P* < 0.001; sensitivity, 72%; specificity, 85%), and 42.5% for 3D-RVEF (AUC, 0.93, *P* < 0.001; sensitivity, 83%; specificity, 96%).

**Figure 3:**
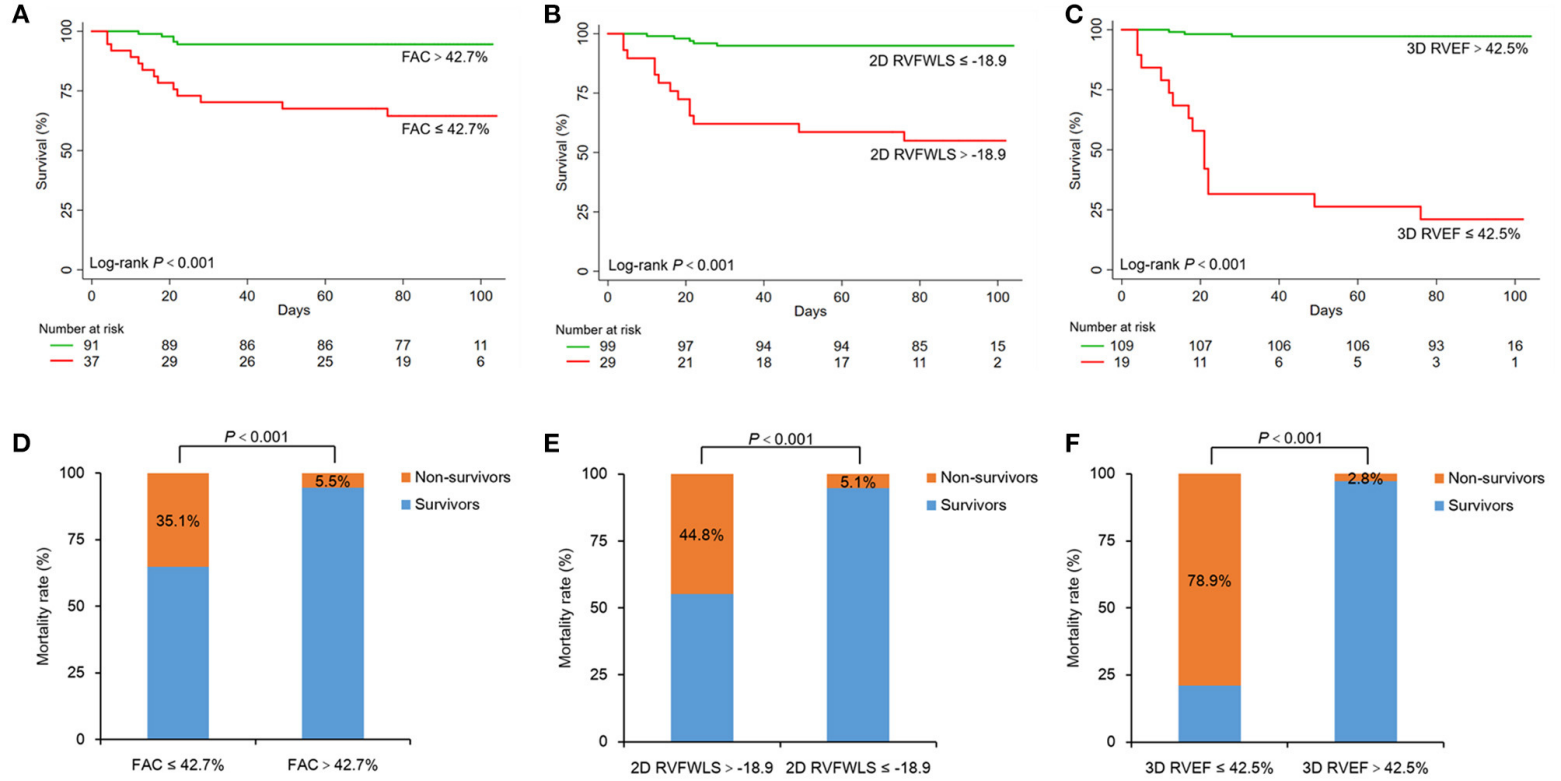
Kaplan–Meier survival curves and percent bar graph of mortality rate in COVID-19 patients. Top, cumulative percentage survival free from death according to FAC **(A)**, 2D-RVFWLS **(B)**, and 3D-RVEF **(C)**. Bottom, mortality rates of different groups dividing by the cut-off value of FAC **(D)**, 2D-RVFWLS **(E)**, and 3D-RVEF **(F)**. COVID-19, coronavirus disease 2019; 2D-RVFWLS, two-dimensional right ventricular free wall longitudinal strain; 3D-RVEF, three-dimensional right ventricular ejection fraction; FAC, fractional area change.

Kaplan–Meier survival curves showed lower survival rates for the groups with decreased RVFAC ( ≤ 42.7%), 2D-RVFWLS (>−18.9%), and 3D-RVEF ( ≤ 42.5%) that was classified by cutoff values of the above RV functional parameters (Figures 3A–C). In addition, decreased RVFAC, 2D-RVFWLS, and 3D-RVEF occurred in 37 (28.9%) patients, 29 (22.7%) patients and 19 (14.8%) patients, respectively. The incidence rate of mortality in these patients was significantly higher than in patients whose RVFAC (>42.7%), 2D-RVFWLS ( ≤ −18.9%), and 3D-RVEF (>42.5%) were not decreased (Figures 3D–F; *P* < 0.001 for all). In addition, we further divided the COVID-19 patients into three subgroups: 3DRVEF > 45% (*n* = 107), 40% < 3DRVEF ≤ 45% (*n* = 15), 30% < 3DRVEF ≤ 40% (*n* = 6). The Kaplan–Meier survival curves showed that the three groups had significantly different survival rates (*P* < 0.001), with the group of 30% < 3DRVEF ≤ 40% having the lowest survival rate (Supplementary Figure 1).

**Figure 4:**
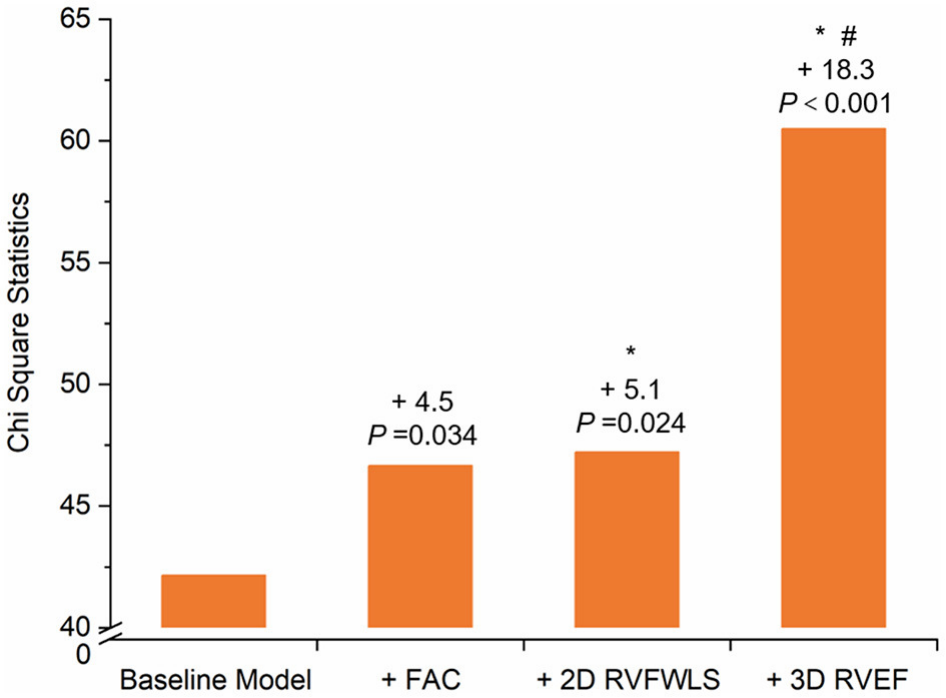
Chi-Square Statistic of Models. Chi-square statistic of the baseline model and different RV functional tests value to predict mortality over the baseline model. Values indicate the additional chi-square value of the different models. *P < 0.05 vs. FAC and ^#^*P* < 0.001 vs. 2D-RVFWLS. Statistical comparisons by likelihood ratio tests. 2D-RVFWLS, two-dimensional right ventricular free wall longitudinal strain; 3D-RVEF, three-dimensional right ventricular ejection fraction; FAC, fractional area change; RV, right ventricular.

In univariate analysis (Table 5), sex, acute cardiac injury, ARDS, RVFAC, 2D-RVFWLS, and 3D-RVEF were significantly associated with higher mortality in COVID-19 patients. In stepwise multivariate analysis, acute cardiac injury and ARDS were used to construct the baseline model for predicting death in COVID-19 patients. Separated models using RVFAC, 2D-RVFWLS, and 3D-RVEF were found to have significant additional prognostic value for mortality over the baseline model (Table 4, Figure 4). Notably, the incremental predictive value of 3D-RVEF (chi-square to improve 18.3; *P* < 0.001) was significantly higher (*P* < 0.05) than RVFAC (chi-square to improve 4.5; *P* = 0.034) and 2D-RVFWLS (chi-square to improve 5.1; *P* = 0.024).

**Table 5:** Univariate and multivariate COX proportional hazard models for predicting death of COVID-19 patients.

	**Univariate analysis**		**Multivariate analysis**							
			**Baseline model 1**		**Model 2 with RVFAC**		**Model 3 with RVFWLS**		**Model 4 with 3DRVEF**	
	**HR (95% CI)**	***P-*value**	**HR (95% CI)**	***P*-value**	**HR (95% CI)**	***P*-value**	**HR (95% CI)**	***P*-value**	**HR (95% CI)**	***P*-value**
Age > 65 years	1.862 (0.735, 4.719)	0.190								
Male	3.164 (1.128, 8.877)	0.029								
Hypertension	1.883 (0.743, 4.771)	0.182								
Diabetes mellitus	0.722 (0.166, 3.142	0.665								
Cardiac disease	2.578 (0.919, 7.234)	0.072								
COPD	2.573 (0.591, 11.199)	0.208								
Malignancy	1.789 (0.411, 7.786)	0.438								
D-dimer, mg/L	1.106 (0.961, 1.272)	0.159								
Acute cardiac injury	7.119 (2.756, 18.387)	<0.001	5.410 (2.084, 14.047)	0.001	3.981 (1.472, 10.765)	0.006	3.209 (1.129, 9.120)	0.029	3.223 (1.230, 8.446)	0.017
ARDS	33.437 (4.446, 251.447)	<0.001	28.102 (3.721, 212.250)	0.001	17.994 (2.302, 140.660)	0.006	17.550 (2.229, 138.179)	0.007	9.404 (1.119, 79.064)	0.039
LVEF, %^*^	1.045 (0.964, 1.133)	0.288								
TAPSE, mm^*^	0.959 (0.849, 1.083)	0.498								
S', cm/s^*^	1.130 (0.973, 1.313)	0.108								
PH	7.564 (2.990, 19.136)	<0.001								
RVFAC, %^*^	0.794 (0.710, 0.889)	<0.001			0.874 (0.768, 0.996)	0.043				
RVFWLS, %^*^	1.401 (1.202, 1.633)	<0.001					1.180 (1.008, 1.381)	0.039		
RVEF, %^*^	0.761 (0.705, 0.822)	<0.001							0.809 (0.735, 0.889)	<0.001

* *Per 1 unit increase. ARDS, acute respiratory distress syndrome; CI, confidence interval; COPD, chronic obstructive pulmonary disease; COVID-19, coronavirus disease 2019; LVEF, left ventricular ejection fraction; HR, hazard ratio; PH, pulmonary hypertension; TAPSE, tricuspid annular plane systolic excursion; RVFAC, right ventricular fractional area change; RVFWLS, right ventricular free wall longitudinal strain; RVEF, right ventricular ejection fraction*.

### Variability of 2D-STE and 3DE Measurements

The intraobserver and interobserver variability for RVFWLS were 0.3 ± 4.3% and 0.6 ± 5.8%, 3D-RVEF were 0.3 ± 3.1% and 0.5 ± 3.9%. The intraobserver and interobserver ICC for RVFWLS were 0.95 and 0.90, 3D-RVEF were 0.95 and 0.91.

## Discussion

To our knowledge, this is the first study to comprehensively depict the conventional, 2D strain and 3DE characteristics of RV in COVID-19 patients with different severity of illness and to explore the prognostic value of 3D-RVEF in COVID-19 patients by directly comparing its utility with that derived from conventional echocardiography and 2D-STE. The major findings were as follows: (1) critical COVID-19 patients were more prone to have larger right heart chamber size, more impaired RV function and a higher prevalence of PH; (2) RVFAC, 2D-RVFWLS, and 3D-RVEF were all significant predictors for mortality in COVID-19 patients; and (3) 3D-RVEF could provide incremental value over 2D-RVFWLS and conventional echocardiographic parameters for predicting mortality in COVID-19 patients.

### RV Size and Function in COVID-19 Patients

Accumulating studies revealed that acute cardiac injury was a common complication and was associated with fatal outcomes in COVID-19 patients (1, 2, 5). We found 27 (21.1%) patients in this cohort had acute cardiac injury as determined by plasma hs-TNI levels. The increased cardiac stress due to respiratory failure and hypoxemia may contribute to cardiac injury and the RV may bear the brunt of its impact (3, 25). Therefore, assessment of RV structure and function could be imperative and significant for COVID-19 patients. There are certain limitations for the assessment of RV size and function by 2D echocardiography due to its complex geometrical anatomy. 3D analysis has the advantage of full-volume acquisition of the entire RV, which may overcome the limitations of 2D analysis. In this study, we assessed RV size and function by the novel, fully automated 3D RV quantification software based on new machine learning algorithm, which provided reasonably accurate RV function measurements are available for clinical use with excellent reproducibility and reliability, as well as less analysis time (10).

Our study showed that COVID-19 patients and the controls had similar size of right heart chambers, which was consistent with a previous study (9). We further depicted the right heart chamber size in COVID-19 patients with different severity of illness and found that the critical groups had larger right heart chambers than general and severe groups. Worse RVFAC, RVFWLS, and 3D-RVEF were also noted in COVID-19 patients than in controls. Moreover, decreased RV systolic function was more marked in critical patients and less pronounced in general and severe groups. A previous study has pointed out that severe COVID-19 patients might progress to ARDS more quickly (26). ARDS might cause a rise in RV afterload by increased vascular resistance and hypoxemia (3). The proportion of ARDS in critical groups was significantly higher than general and severe groups in our study, which may explain why critical groups were more likely to had the larger right heart chambers and RV dysfunction. It is suggested that clinicians should be alert to RV dysfunction in critically ill patients and take prompt treatments to improve patient outcomes.

### Prognosis of RV Function in COVID-19 Patients

Previously, the prognostic value of RVFWLS and conventional RV function parameters in COVID-19 patients have been reported (9). 3D-RVEF also has been demonstrated as a strong prognostic value in other various cardiovascular diseases (11, 12, 27, 28), while its prognostic value in COVID-19 patients has not been validated yet. In our study, univariate and multivariate regression models revealed 3D-RVEF, RVFWLS, and FAC all were independent predictors for mortality after adjustment for gender, ARDS, and acute cardiac injury. The S′ and TAPSE were not predictors of mortality in our patients, possibly because they are angle-dependent and only reflect the longitudinal function of the basal portion of the RV free wall. RVFAC [cut-off value of 39% by Houard et al. (29) 40% by Amano et al. (30)], RVFWLS [cut-off value of −19% by Houard et al. (29) 22% by Gavazzoni et al. (31)] and 3D-RVEF [cut-off value of 43% by Jone et al. (28)] have been proven to be independent predictors of adverse outcomes in other various cardiovascular diseases. Moreover, a recent study suggested that a 43.5% threshold of RVFAC could help identify COVID-19 patients at higher risks of mortality (9). The prognostic value of RVFAC, RVFWLS, and 3D-RVEF to predict mortality was also noted in our study, with the best cut-off value of 42.7% for RVFAC, −18.9% for RVFWLS, and 42.5% for 3D-RVEF. More notably, we found the multivariate regression model with 3D-RVEF showed an incremental prognostic value of higher mortality over that with RVFWLS and FAC, which was in line with the previous study that reported 3D-RVEF was superior to RVFWLS and conventional echocardiographic parameters in predicting adverse clinical events in PH (28). Additionally, the COVID-19 patients were divided into three subgroups based on the published reference: (16) 3DRVEF > 45%, 40% < 3DRVEF ≤ 45%, and 30% < 3DRVEF ≤ 40%. The Kaplan–Meier survival curves showed that the three groups had significantly different prognosis (*P* < 0.001), with the group of 30% < 3DRVEF ≤ 40% having the lowest survival rate. RVFAC was measured by planimetry of the RV cavity and its measurement variability was limited by the accurate identification of the RV endocardial border. RVFWLS is mainly based on longitudinal myocardium deformation of RV outflow portions, neglecting the contributions of myocardium deformation in other directions (32). The study by Bleakley et al. reported that RVFWLS was not sensitive in identifying RV dysfunction, because severe COVID-19 is associated with a specific phenotype of RV radial impairment with sparing of longitudinal function (33). However, 3D-RVEF can comprehensively evaluate the different parts of the RV (including the inflow, apical, and outflow) and is not limited to longitudinal myocardial function (34, 35). Our study demonstrated that 3D-RVEF as a more robust prognostic indicator for mortality and could provide incremental prognostic value over RVFWLS and conventional echocardiography in COVID-19 patients.

### Clinical Implications

Our findings emphasized that the significance of evaluating RV function and validated its predictive value in COVID-19 patients. Critical COVID-19 patients were more likely to suffer from RV dysfunction. This study offered the first evidence about the prognostic value of RVEF measured by 3DE in COVID-19 patients. 3D-RVEF is theoretically superior to conventional echocardiographic parameters and RVFWLS derived from 2D-STE in assessing RV function due to the complex anatomy of RV. Therefore, we demonstrated that 3D-RVEF could provide an incremental predictive value of death over the RVFWLS and conventional echocardiographic parameters in COVID-19 patients, which may help identify COVID-19 patients at higher risks of adverse outcomes.

### Limitation

Our study did have some limitations. First, as both 3DE and 2D-STE analyses were dependent on good image quality, we excluded 38 (22.1%) patients with insufficient image quality or arrhythmia during examination, which may cause some selection bias. As a result, our findings were not applicable to COVID-19 patients with arrhythmia or unsatisfactory image quality. Moreover, part of subjects (78/128) in our study were included in the previous work (9), which was focus on the prognostic value of RV free wall longitudinal strain (RVFWLS) in COVID-19 patients. Second, this was a single-center study with a relatively small sample of hospitalized COVID-19 patients at different disease status, further studies with multi-center and larger sample size should be performed to validate our findings. Third, the cutoff values reported in this study may not be applicable to other software due to inter-vendor variability. Finally, the current fully automated 3D RV software does not provide 3D RV strain values yet, and hence the evidence of the prognostic value of 3D RV strain in COVID-19 patients was lacking in our study. Future studies should be performed to determine the prognostic superiority of 3D RV strain.

### Conclusions

Our study emphasized that 3D-RVEF was an independent predictor of mortality in COVID-19 patients and provided an incremental prognostic value superior to RVFWLS and conventional echocardiographic parameters.

## Data Availability Statement

The original contributions presented in the study are included in the article/Supplementary Material, further inquiries can be directed to the corresponding author/s.

## Ethics Statement

The studies involving human participants were reviewed and approved by the Ethics Committee of Tongji Medical College, Huazhong University of Science and Technology. Written informed consent was waived for all participants with emerging infectious diseases. Written informed consent for participation was not required for this study in accordance with the national legislation and the institutional requirements. Written informed consent was not obtained from the individual(s) for the publication of any potentially identifiable images or data included in this article.

## Author Contributions

All authors listed have made a substantial, direct and intellectual contribution to the work, and approved it for publication.

## Conflict of Interest

The authors declare that the research was conducted in the absence of any commercial or financial relationships that could be construed as a potential conflict of interest.

## Abbreviations

2D: Two-dimensional
3D: Three-dimensional
3DE: Three-dimensional echocardiography
A: Late diastolic inflow velocity
COVID-19: Coronavirus disease 2019
E: Early diastolic inflow velocity
e': Early diastolic tissue velocity
FAC: Fractional area change
hs-TNI: high-sensitivity troponin I
ICC: intra-class correlation coefficient
IQR: interquartile range
PH: Pulmonary hypertension
RVFWLS: right ventricular free wall longitudinal strain
RVEDVI: Right ventricular end-diastolic volume index
RVESVI: Right ventricular end-systolic volume index
SARS-CoV-2: Severe acute respiratory syndrome coronavirus 2
STE: Speckle-tracking echocardiography
S': Tricuspid lateral annular systolic velocity
TAPSE: Tricuspid annular plane systolic excursion
TR: Tricuspid regurgitation.
