# Supplementary figures and images for: Prognostic Value of Right Ventricular Ejection Fraction Assessed by 3D Echocardiography in COVID-19 Patients
Source: Front Cardiovasc Med. 2021 Feb 9;8:641088. doi: 10.3389/fcvm.2021.641088 (PMC7902006; doi:10.3389/fcvm.2021.641088)

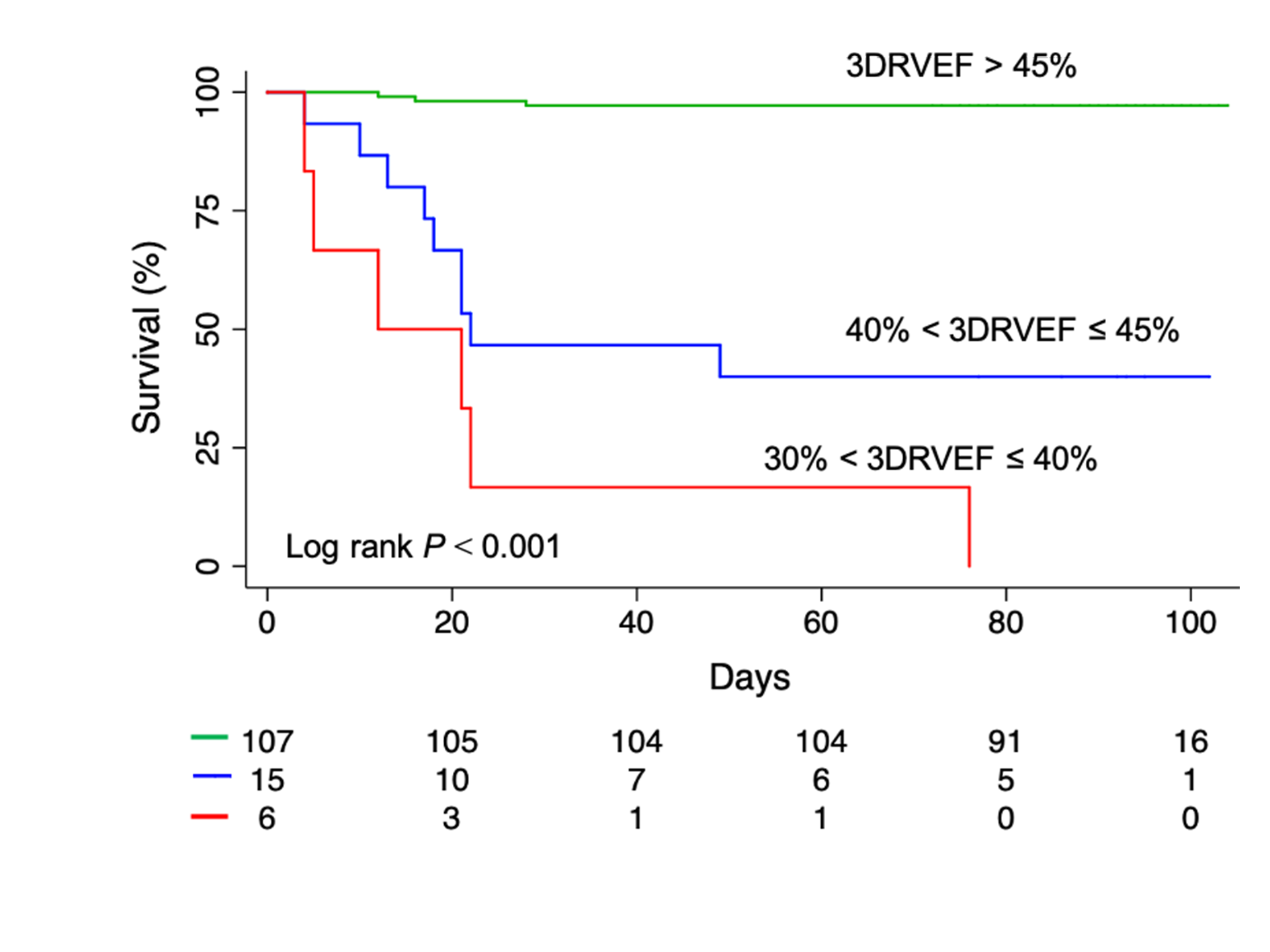

Supplement: Supplementary file 1 [file Image_1.TIF]
