# Supplementary material for: Prognostic Value of Right Ventricular Ejection Fraction Assessed by 3D Echocardiography in COVID-19 Patients
Source: Front Cardiovasc Med. 2021 Feb 9;8:641088. doi: 10.3389/fcvm.2021.641088 (PMC7902006; doi:10.3389/fcvm.2021.641088)
Supplement: Supplementary file 2 [file Presentation_1.pdf]

Supplemental Material

Supplemental Figure 1: **Kaplan-Meier survival curves according to the severity of right ventricular dysfunction in COVID-19 patients.**

Patients with different severity of right ventricular dysfunction had significantly different survival rates ($P<0.001$).

COVID-19, coronavirus disease 2019; 3D-RVEF, three-dimensional right ventricular ejection fraction.
